# Le carcinome lobulaire infiltrant du sein: à propos de 30 cas

**DOI:** 10.11604/pamj.2019.34.70.18780

**Published:** 2019-10-04

**Authors:** Bergaoui Haïfa, Houda El Mhabrech, Inès Zouari, Manel Njima, Amira Daldoul, Hajji Ahmed, Hajjaji Awatef, Wiem Khchine, Sonia Zaidi, Raja Faleh

**Affiliations:** 1 Service de Gynécologie Obstétrique, Centre de Maternité et Néonatologie de Monastir, Tunisie; 2 Service de Radiologie, Hadj Ali Soua Ksar Hellal, Tunisie; 3 Service d'Anatomopathologie, Eps Fattouma Bourguiba Monastir, Tunisie; 4 Unité de Carcinologie, Centre de Maternité et Néonatologie de Monastir, Tunisie

**Keywords:** Carcinome lobulaire infiltrant, épidémiologie, traitement, pronostic, Invasive lobular carcinoma, epidemiology, treatment, prognosis

## Abstract

Le cancer lobulaire infiltrant (CLI) du sein représente 5 à 15% des cancers du sein, il est classé le deuxième type histologique le plus fréquent, après le cancer infiltrant de type non spécifique (CINS) et son incidence est en nette augmentation. Son diagnostic clinique et radiologique est difficile. Il est plus fréquemment bilatéral. C'est un cancer d'évolution plus lente que le CINS avec de meilleurs facteurs pronostiques notre étude vise à détailler les caractéristiques cliniques, radiologiques, thérapeutiques et pronostiques du CLI. Il s'agit d'une étude rétrospective descriptive de 30 cas de CLI du sein colligé au Centre de Maternité et Néonatologie Monastir sur une période de 10 ans. L'incidence du CLI était de 5,2%. L'âge moyen est de 53,43 ans. Dix pourcent avaient des antécédents personnels de mastopathies bénignes, 6,66% avaient des antécédents personnels du cancer du sein et 3,33% avaient des antécédents familiaux du cancer du sein. Le CLI était diagnostiqué à un stade tardif chez 18 cas. La masse était bifocale chez 5 patientes, multifocale chez 4 patientes et bilatérale chez 3 patientes. Une seule patiente avait présenté une métastase hépatique lors du diagnostic. La chirurgie radicale type Patey était réalisée d'emblée chez 63,33% des patientes. Des lésions multifocales étaient détectées dans 44,80% des cas lors de l'examen anatomopathologique. Le curage ganglionnaire était positif chez 21 patientes. Vingt-huit patientes soit 93,33% avaient bénéficié d'une radiothérapie et d'hormonothérapie adjuvante. La survie globale à 5 ans était estimée à 77,3%.

## Abstract (en)

Invasive lobular carcinoma (ILC) of the breast accounts for 5-15% of breast cancers. It is classified as the second most frequent histological type after non-specific invasive cancer (NSIC) and its prevalence has seen a marked increase. Clinical and radiological diagnosis is difficult. Usually, this cancer develops bilaterally and more slowly than non-specific invasive cancer, with best prognostic factors. The purpose of our study was to describe in detail the clinical, radiological, therapeutic and prognostic features of invasive lobular carcinoma. We conducted a retrospective descriptive study of 30 cases with ILC of the breast whose data were collected at the Center of Maternity and Neonatology, Monastir over a period of 10 years. The prevalence of ILC was 5.2%. The average age of patients was 53.43 years. Ten percent of patients had a personal history of benign mastopathies, 6.66% had a personal history of breast cancer and 3.33% had a family history of breast cancer. Late-stage diagnosis was made in 18 cases. Bifocal mass was found in 5 patients, multifocal mass in 4 patients and bilateral mass in 3 patients. Only one patient had liver metastasis at the time of diagnosis. Radical mastectomy (Patey) was immediately performed in 63.33% of patients. Anatomo-pathological examination showed multifocal lesions in 44.80% of cases. Lymph node dissection was satisfactory in 21 patients; 28 patients (93.33%) underwent radiotherapy and adjuvant hormonal therapy. Five-year overall survival was 77.3%.

## Introduction

Le carcinome lobulaire infiltrant (CLI) du sein représente 5 à15% des cancers du sein [1, 2], il est classé le deuxième type histologique le plus fréquent, après le cancer infiltrant de type non spécifique (CINS). Son incidence est en nette croissance [3]. Cette augmentation semble être liée à la fréquence de l'utilisation du traitement hormonal substitutif après la ménopause qui pourrait multiplier le risque de développer cette affection par 2 à 3 et de manière beaucoup plus importante que pour le carcinome infiltrant de type non spécifique [4]. La contraception orale et la consommation d'alcool pourraient également augmenter le risque de cancer lobulaire [5, 6]. Le CLI est souvent associé à une atteinte mammaire multifocale et bilatérale. Il métastase préférentiellement au niveau des séreuses et plus particulièrement au niveau péritonéal. Les patientes se présentent généralement dans un stade relativement avancé au moment du diagnostic. Sa prise en charge thérapeutique et son pronostic sont cependant quasi identique à celui du carcinome infiltrant de type non spécifique. Vue la fréquence des récepteurs hormonaux au niveau de la masse tumorale, l'hormonothérapie est indispensable dans l'arsenal thérapeutique.

## Méthodes

Etude rétrospective descriptive de 30 observations de CLI du sein colligées au Service de Gynécologie Obstétrique du Centre de Maternité et Néonatologie de Monastir sur une période de 10 ans allant du 01 janvier 2008 jusqu'au 31 décembre 2017. Les critères d'inclusion étaient toutes les patientes prises en charge dans notre service pour CLI durant la période d'étude. Les critères d'exclusion étaient les tumeurs mammaires sans preuves histologiques et les patientes non prises en charge dans notre service. Les caractéristiques étudiées sont épidémiologiques, cliniques, radiologiques, histologiques et la conduite thérapeutique

## Résultats

**Figure 1:**
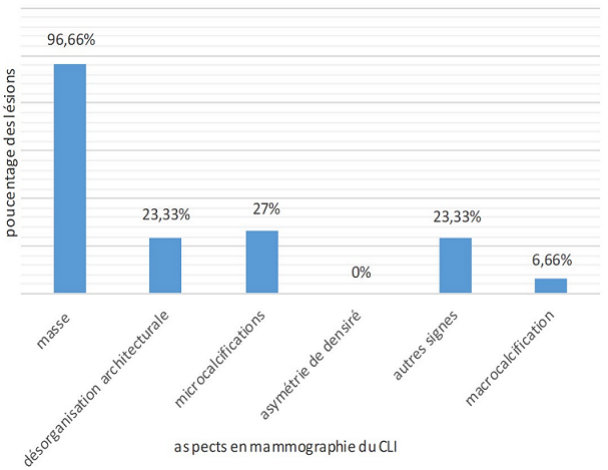
Aspects mammographiques du CLI

**Figure 2:**
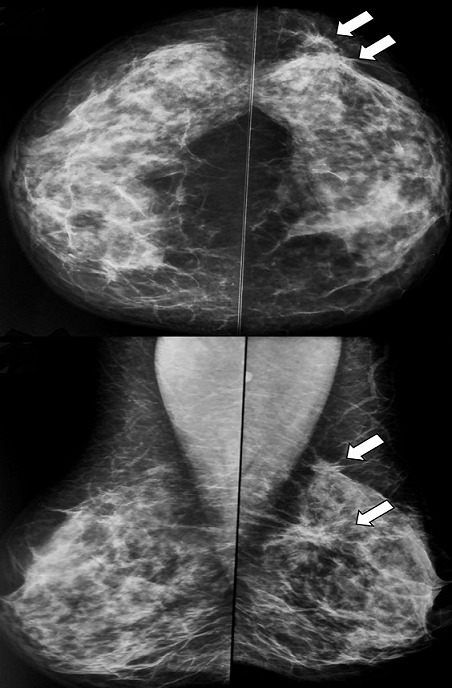
Mammographie bilatérale en incidence de face; (A) et d’oblique externe (B): seins de densité hétérogène type « c » de BIRADS. 2 Masses à contours stellaires siège au niveau du QSEG (flèches épaisses). Le sein droit est d’aspect normal. Absence d’adénopathies axillaire

**Figure 3:**
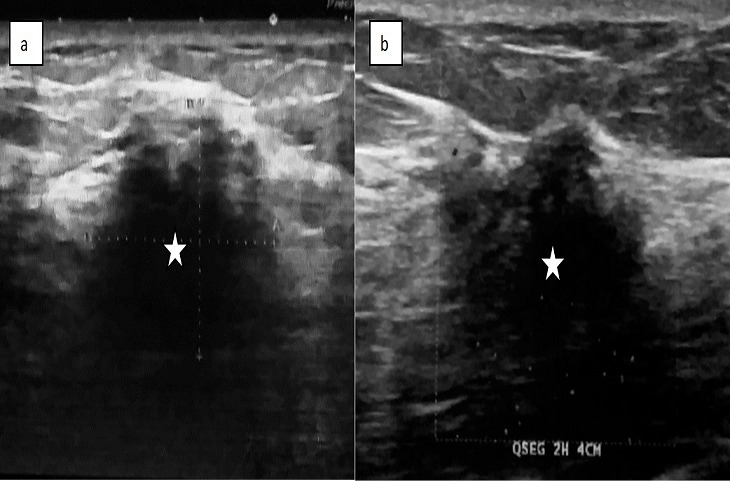
Échographie mammaire (A,B): masse hypoéchogène (étoile) à grand axe Perpendiculaire à la peau, s’accompagnant d’atténuation des échos en postérieur

**Figure 4:**
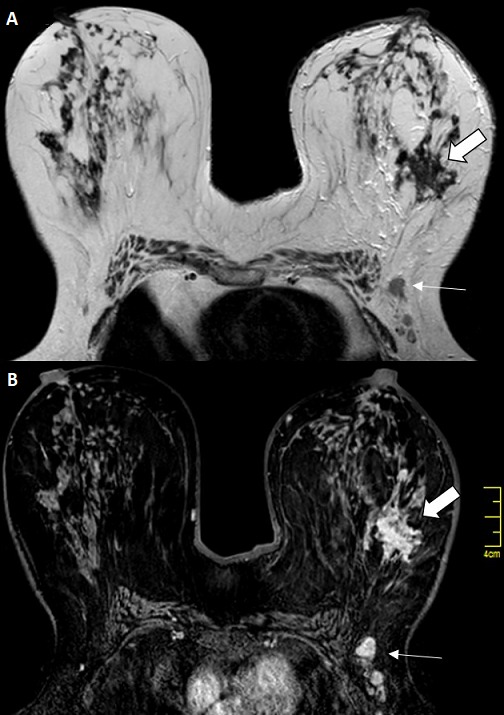
IRM mammaire en coupe axiale en SE T2 (A) et en SE T1 après injection dynamique de Gadolinium; (B) masse à contours stellaire du QSE gauche (flèche épaisse) s’accompagnant d’un rehaussement de type non masse. Adénopathies axillaires gauches (flèches)

L'incidence du CLI dans notre étude était de 4%, elle passait de 2% en 2008 à 7% en 2017. Il touchait la tranche d'âge comprise entre 50 et 60 ans, avec un âge moyen de 53,43 ans. Vingt-sept patientes dans notre série sont multipares soit 90%. Seize patientes soit 53,33% sont ménopausées lors du diagnostic. Dix pour cent avaient des antécédents personnels des mastopathies bénignes, 6,66% avaient des antécédents personnels du cancer du sein et 3,33% avaient des antécédents familiaux du cancer du sein. Le CLI était diagnostiqué à un stade tardif (après 6 mois) chez 18 cas, soit 60%. Le signe révélateur le plus fréquent était un nodule chez 25 cas soit 83,33% avec une taille moyenne de 3,31cm. Sur le plan radiologique: le CLI se manifestait le plus souvent par une masse spiculée à la mammographie (74,35%) (Figure 1, Figure 2) et une image hypoéchogène non circonscrite à contours irréguliers à l'échographie (84,6%) (Figure 3). La masse était bifocale chez 5 patientes soit 16,66%, multifocale chez 4 patientes soit 13,33% et bilatérale chez 3 patientes soit 10%. L' imagerie par résonance magnétique (IRM) était indiquée chez 3 patientes et avait objectivé un rehaussement de type non masse chez 2 patiente (Figure 4). Une seule patiente avait présenté une métastase hépatique lors du diagnostic tandis que le bilan d extension était négatif chez les restes des patientes. La majorité des tumeurs étaient classées T2 (44,44%). La chirurgie radicale type Patey était réalisée d'emblée chez 63,33% des patientes tandis que dans16,66% des cas avaient bénéficié d'un traitement conservateur et 20% avaient subi une mastectomie de rattrapage. Des lésions multifocales étaient détectées dans 44,80% des cas lors de l'examen anatomopathologique de la pièce opératoire. L'atteinte ganglionnaire était confirmée chez 36,66% soit 11 patientes. La majorité des tumeurs étaient classé SBR2 (73,33% des cas). Des récepteurs hormonaux ostrogéniques et progestatifs étaient positifs respectivement dans 93% et 86,20 % des cas. Une surexpression du gène HER2 étaient révélé seulement chez 4 cas soit 14%. Une chimiothérapie néoadjuvante a été délivrée pour 4 patientes (13,33%) et une chimiothérapie adjuvante pour 23 cas (76,66%). Vingt-huit patientes soit 93,33% avaient bénéficié d'une radiothérapie et d'hormonothérapie adjuvante. La survie globale à 5ans était estimée à 77,3%.

## Discussion

Actuellement le cancer du sein représente le premier cancer de la femme dans le monde, dominé par le cancer invasif qui représente plus de 90% de l'ensemble de ses types histologiques [1]. Parmi les carcinomes invasifs, le CLI demeure particulier à cause de sa difficulté diagnostique et son mode de prolifération. Son incidence est en nette croissance, passant de 9,5 % en 1987 à 15,6 % en 1999 aux États-Unis, des hypothèses ont attribué cette hausse à l'augmentation de la prescription de traitement hormonal substitutif et la contraception orale [2]. Ce résultat a été également démontré dans notre étude avec une augmentation de la fréquence du CLI allant de 2% en 2008 pour atteindre le 7% en 2017. L'évolution et les développements des moyens du diagnostic tels que l'échographie et l'IRM qui sont plus sensibles que la mammographie dans la détection des CLI [3], ainsi que l'amélioration des techniques histopathologiques, peuvent également contribuer à l'augmentation du taux de détection des CLI, expliquant ainsi son incidence croissante ces dernières années. Le CLI touche surtout les femmes âgées, souvent ménopausées, et comparativement au CINS, il survient chez des femmes généralement plus âgées avec un écart de 3 ans [4]. Dans notre série, l´âge moyen de survenue du CLI était de 53,43 ans avec des extrêmes allant de 40 à 79 ans et la tranche d´âge entre 40 et 50 ans était la plus touchée. Plusieurs études ont démontré qu'il existe un lien de causalité entre le traitement hormonal substitutif et la survenue du CLI puisque ceci augmente le risque de cette affection par 2 à 3 et de manière beaucoup plus importante que pour le CINS [5]. Reeves *et al.* ont retrouvé un risque relatif à l´utilisation des THS après la ménopause de 2,25 (IC: 2,00-2,52) pour les cancers lobulaires et de 1,63 (1,55-1,72) pour les cancers canalaire [6]. Selon notre étude cette hypothèse n'a pas été étayée car aucune de nos patientes n'a bénéficié d'un traitement hormonal substitutif. Certaines tumeurs bénignes du sein à type de mastopathie fibrokystique ou hyperplasie lobulaire et surtout les mastopathie prolifératives et atypiques contribuent à accroitre le risque du cancer du sein. Dans notre série, seulement 03 patientes ont rapporté des antécédents personnels de mastopathie bénigne qui représentait 10% de l'ensemble des cas étudiés. Certainement le facteur génétique intervient et augmente le risque de survenue du CLI puisqu'il est lié à plusieurs mutations, entre autres la mutation CDH1 qui est la plus fréquente dans le cas du CLI [7]. De même la mutation du gène BRCA2 situé sur le chromosome 13 et la mutation BRCA1 sur le chromosome 17 augmente respectivement la fréquence de survenue du CLI de 8,4% et 2,2 % [8]. Dans notre série seulement une seule patiente (3,33%) avait une histoire familiale de cancer du sein.

Le CLI évolue souvent d'une manière quiescente : un délai de consultation qui dépasse les 6 mois est généralement démontré dans plusieurs études [9]. Quant à notre étude, 60% des patientes ont consulté après 6 mois. Selon plusieurs études, le CLI se localise préférentiellement au niveau du sein gauche. Selon Wasfi *et al.* [10], la tumeur se trouve au sein gauche dans 50,9% des cas. Dans notre série, conformément aux données de la littérature, 62% des tumeurs sont du côté gauche. La mammographie présente une sensibilité faible (57 à 79%) dans la détection du CLI. Ceci a suscité de l'intérêt pour d´autres modalités d´imagerie, telles que l´échographie, l´imagerie par résonance magnétique, la tomosynthèse et l´imagerie moléculaire ciblée [11]. La sensibilité de l'échographie dans la détection du CLI varie entre 68% et 98%. Un taux de faux négatif peut aller jusqu'à 12% des cas [12]. L'échographie est supérieure à la mammographie dans la détection de la multicentricité et de la multifocalité dont la sensibilité est d'environ 21% dans la série de Selinko [13]. Ces études renforcent le fait que, dans le contexte de résultat d´examen physique suspect combiné à une mammographie «normale», l'échographie constitue un ajout très précieux dans le diagnostic du CLI. Dans notre série, l'échographie mammaire a été combinée à la mammographie chez toutes les patientes. Elle a montré une image hypoéchogène non circonscrite dans 84,61% associée à des images d'atténuation acoustique postérieure dans 51,72% des cas. L'IRM s'est imposée comme l'examen indispensable dans le bilan d'extension locale du CLI [14]. En effet, plusieurs études ont confirmé la haute sensibilité de l'IRM par rapport aux autres examens radiologiques dans l'évaluation de l'extension tumorale locorégionale. La majorité des lésions se traduisent à l'IRM par des rehaussements type masse avec des caractéristiques typiques de malignité. Selon les recommandations, un bilan d'extension d'un CLI doit comporter [15]: une radiographie thoracique, une échographie hépatique, une scintigraphie osseuse (pour les tumeurs de plus de 1cm).

L'originalité du CLI réside dans son mode de dissémination métastatique. Selon Chann *et al.* le CLI peut s'étendre vers des sites inhabituels : péritoine, rétro péritoine et viscères creux. Dans l'ensemble, le CLI se caractérise par une infiltration diffuse de ces organes, similaire aux lymphomes. Ces localisations particulières s'observent tardivement et peuvent passer inaperçus cliniquement [16]. L'objectif de ce bilan est la détection des métastases susceptibles de modifier l'attitude thérapeutique. Pour le CLI, les formes métastatiques d'emblée sont rares. Selon Fondriner *et al.* [17] les formes métastatiques d'emblée ont été retrouvées dans 0,5% des cas. Dans notre série, une seule patiente a consulté au stade métastatique (localisation hépatique) ce qui représentait 3,33%. Les modalités thérapeutiques du CLI du sein diffèrent en fonction de la taille, du stade, de la présence ou non des métastases et des facteurs histo-pronostique de la tumeur. L'examen anatomo-pathologique des pièces opératoires permet d'une part de poser le diagnostic de certitude du type histologique du cancer de sein, et d'autre part d´établir la classification TNM finale afin d'orienter la prise en charge thérapeutique et prédire le pronostic de la maladie. Le traitement du cancer du sein se base sur deux volets: traitement locorégional: résection de la tumeur primitive et les territoires ganglionnaires de drainage. Traitement général: traiter une éventuelle dissémination infra-clinique à distance par radiothérapie qui diminue significativement le risque de récidive locale à 5 ans: d'après l'étude de Diepenmaat *et al.* [18], la récidive n´était que de 2,1%. La chimiothérapie néo-adjuvante plusieurs études affirment que les CLI ne sont pas sensibles à la chimiothérapie néo adjuvante et donc on a recours plutôt à la chimiothérapie adjuvante. L'hormonothérapie, le CLI est une tumeur hormono sensible. Dans la littérature, plus de 90% des CLI possèdent des récepteurs hormonaux positifs, alors que seulement 5-14% des CLI sont cliniquement HER-2positif [19]. La thérapie ciblée l'étude de Reis-Filho [20] a identifié une amplification du FGFR1 au cours du CLI, en suggérant l'étude de nouvelles molécules ou anticorps dirigés contre le FGFR1. Dans notre série 13,33% avaient bénéficié de la thérapie ciblée. L'évolution des CLI est le plus souvent favorable une fois traitée convenablement. Dans notre étude la survie globale à 10 était estimée à 63,3%.

## Conclusion

Le CLI est une variété particulière et rare de cancer du sein dont l'incidence est en nette augmentation durant ces dernières années. Nous avons trouvé des caractéristiques cliniques et radiologiques variées et concordaient en grande partie avec la littérature. On insiste sur la place d'IRM qui occupait une place croissante dans la prise en charge du CLI, en raison de sa supériorité prouvée par rapport à la mammographie et à l'échographie en terme de détection de multifocalité et multicentricité.

### Etat des connaissances actuelles sur le sujet

- Le CLI se développe chez les femmes âgées, souvent ménopausées, consultant pour la découverte d'une masse mammaire;
- Le CLI est caractérisé par sa multifocalité et sa bilatéralité.

### Contribution de notre étude à la connaissance

- Cette étude permettra de montrer l'ampleur de ce type de cancer dans un pays nord-africain dépourvu de politique de dépistage;
- La connaissance des différents aspects diagnostiques du CLI permettra d'améliorer sa prise en charge.

## Conflits des intérêts

Les auteurs ne déclarent aucun conflit d’intérêts.
